# Characterizing Emergency Department Care for Patients With Histories of Incarceration

**DOI:** 10.1016/j.acepjo.2024.100022

**Published:** 2025-01-10

**Authors:** Thomas Huang, Vimig Socrates, Polina Ovchinnikova, Isaac Faustino, Anusha Kumar, Conrad Safranek, Ling Chi, Emily A. Wang, Lisa Puglisi, Ambrose H. Wong, Karen H. Wang, R. Andrew Taylor

**Affiliations:** 1 Department of Emergency Medicine, Yale School of Medicine, New Haven, Connecticut, USA; 2 Department for Biomedical Informatics and Data Science, Yale University School of Medicine, New Haven, Connecticut, USA; 3 Program of Computational Biology and Bioinformatics, Yale University, New Haven, Connecticut, USA; 4 SEICHE Center for Health and Justice, Yale School of Medicine, New Haven, Connecticut, USA; 5 Equity Research and Innovation Center, Yale School of Medicine, Yale University, New Haven, Connecticut, USA

**Keywords:** incarceration, emergency medicine, large language models, artificial intelligence, quality of health care

## Abstract

**Objectives:**

Patients with a history of incarceration experience bias from health care team members, barriers to privacy, and a multitude of health care disparities. We aimed to assess care processes delivered in emergency departments (EDs) for people with histories of incarceration.

**Methods:**

We utilized a fine-tuned large language model to identify patient incarceration status from 480,374 notes from the ED setting. We compared socio-demographic characteristics, comorbidities, and care processes, including disposition, restraint use, and sedation, between individuals with and without a history of incarceration. We then conducted multivariable logistic regression to assess the independent correlation of incarceration history and management in the ED while adjusting for demographic characteristics, health behaviors, presentation, and past medical history.

**Results:**

We found 1734 unique patient encounters with a history of incarceration from a total of 177,987 encounters. Patients with history of incarceration were more likely to be male, Black, Hispanic, or other race/ethnicity, currently unemployed or disabled, and had smoking and substance use histories, compared with those without. This cohort demonstrated higher odds of elopement (OR: 3.59 [95% CI: 2.41–5.12]), leaving against medical advice (OR: 2.39 [95% CI: 1.46–3.67]), and being subjected to sedation (OR: 3.89 [95% CI: 3.19–4.70]) and restraint use (OR: 3.76 [95% CI: 3.06–4.57]). After adjusting for covariates, the association between incarceration and elopement remained significant (adjusted odds ratio: 1.65 [95% CI: 1.08–2.43]), while associations with other dispositions, restraint use, and sedation did not persist.

**Conclusion:**

This study identified differences in patient characteristics and care processes in the ED for patients with histories of incarceration and demonstrated the potential of using natural language processing in measuring care processes in populations that are largely hidden, but highly prevalent and subject to discrimination, in the health care system.

**The Bottom Line**

Research addressing healthcare disparities in emergency medicine for patients with a history of incarceration is limited by challenges in identification through electronic health records. This retrospective analysis, leveraging a large language model to examine emergency department notes, identified unique socio-demographic characteristics and disparities in care processes among this population, including higher rates of sedation, physical restraint use, and elopement. These findings highlight systemic challenges faced by this underserved population and provide valuable insights to inform future interventions aimed at reducing bias in emergency care settings.

## 1 Introduction

### 1.1 Background

The United States continues to have one of the highest incarceration rates globally, with over 7.23 million admissions reported annually.^1^ Incarcerated individuals face numerous obstacles as they attempt to reintegrate into society, including barriers to receiving health care and higher rates of chronic health conditions, mental health disorders, substance use disorders, and communicable and noncommunicable diseases.^2^, ^3^, ^4^ The culmination of these factors ultimately leads to an elevated risk of death, hospitalization, and deteriorating health outcomes post-release.^2^^,^^5^, ^6^, ^7^, ^8^

### 1.2 Importance

Despite these issues, few studies have explored care processes for individuals with incarceration histories in the emergency department (ED). Existing studies have mostly focused on characterizing currently incarcerated patients.^9^, ^10^, ^11^ Studies investigating restraint and sedation usage in these populations are also limited by cohort size and focus on subpopulations such as pregnant patients.^12^^,^^13^ Research regarding patients with a history of incarceration remains limited, in part due to current limitations in identifying patients with histories of incarceration using electronic health records (EHRs). Although the judicious use of physical or chemical restraints is intended for the safety of staff and patients, they have been found to be associated with severe psychological distress, may reinforce negative perceptions, and can elicit trauma for patients.^14^, ^15^, ^16^ Recent work by our group employing large language models (LLMs) has enabled more accurate and scalable identification to identify patients with a history of incarceration.^17^ By leveraging LLMs to identify larger cohorts for studies, we hope to be able to better characterize disparities across multiple axes within the hospital to aid health care workers in developing tailored care and systemic interventions to meet the unique needs of patients with histories of incarceration.

### 1.3 Goals of This Investigation

The primary objective of this investigation is to characterize patients with a history of incarceration and the management of their stay in the ED. We hypothesized that those with histories of incarceration will have worse care processes compared with those without a history.

## 2 Methods

### 2.1 Study Design

The study population consisted of a retrospective cohort of adult patients aged (≥18 years) who presented to the ED between January 1, 2022, and December 31, 2022. A total of 177,987 unique patients were identified within this inclusion criteria, with 480,374 corresponding notes. For each ED patient, only the most recent ED encounter was retrieved.

### 2.2 Setting

The study was completed across 10 ED sites within the Yale-New Haven Health System, a regional health care network in the northeastern United States, covering a large geographically diverse area and closely resembling the overall national population.^18^^,^^19^ This study followed the strengthening of the reporting of observational studies in epidemiology reporting guidelines for observational studies and was approved by the institutional review board, which waived the need for informed consent (HIC# 1602017249).

### 2.3 Measures/Outcomes

Our fine-tuned LLM was previously shown to be able to identify the presence of a history of incarceration with high predictive performance.^20^ Our LLM was fine-tuned by utilizing a pretrained BERT-based model Clinical-Longformer and further training it on an annotated subset of rich data for incarceration status based on notes taken from the ED. The total cost to deploy the model in the present study was $0.0875 USD. For the present study, to identify patients with a history of incarceration, we applied the model with a threshold of 95% positive predictive value to the total set of notes that met inclusion criteria (note types and frequencies in Supplementary Appendix A1). The labels assigned to corresponding notes were aggregated within each encounter. These unique encounters and medical record numbers were then linked with a larger ED data set, including demographic data, encounter-specific metrics, and social-behavioral variables that were obtained as an extraction from the EHRs.

Demographic data were collected on age, sex, race/ethnicity, zip code, insurance, and employment status. Historical health information was collected, including substance use history, smoking history, and presence of a primary care provider (PCP). Substance use history was defined by the usage of any substances in Supplementary Appendix A2. To better understand the care processes experienced by the patient during their ED visit, data including disposition, medical restraint usage, and sedation usage in the ED was extracted.^21^, ^22^, ^23^ Chief concern was categorized into 6 overall categories: medical, trauma, agitation, cognitive/neurologic, alcohol/drug, and psychiatric. All data were obtained from the system-wide EHR (Epic, Verona, WI) using a centralized data warehouse (Helix).

### 2.4 Data Analysis

Categoric data, including demographic variables, were described using frequency counts and proportions to their respective cohorts (patients with a history of incarceration vs patients without a history of incarceration). Prior to multivariable logistic regression, a mode imputation was utilized to correct for unknown data fields. A total of 25,959 (0.47%) data points were imputed utilizing this method. To identify an independent association between incarceration history and management practices in the ED, a multivariable logistic regression model was developed with the history of incarceration as the dependent variable and demographic data, historical health information, and metrics from within the ED encounter as covariates. Chi-square statistics were used to calculate odds ratios (ORs).

## 3 Results

Among a total of 177,987 unique patients, 1734 (0.97%) patients were found to have a history of incarceration by natural language processing identification. Patients presenting to the EDs with prior incarceration were predominantly male (77.5% vs 43.3%), younger (aged 44.3 vs 51.9 years), Black (39.3% vs 18.6%), unemployed (57.2% unemployed vs 19.3%), and living with a disability (16% vs 6%) compared with those without incarceration history (Table 1).

**Table 1:** Demographic information of emergency department patients from January 1, 2022 until December 31, 2022, organized by encounter.

Category		Overall	Any Hx of Incarceration	No. Hx of incarceration
N		177,987	1734	176,253
Age group, n (%)	18-24	19020 (10.7)	69 (4.0)	18,951 (10.8)
-	25-34	27,476 (15.4)	393 (22.7)	27,083 (15.4)
-	35-44	26,605 (14.9)	461 (26.6)	26,144 (14.8)
-	45-54	23,813 (13.4)	393 (22.7)	23,420 (13.3)
-	55-64	27,693 (15.6)	337 (19.4)	27,356 (15.5)
-	65+	53,358 (30.0)	81 (4.7)	53,277 (30.2)
-	Unknown	22 (0.0)		22 (0.0)
Sex, n (%)	Female	100,292 (56.3)	390 (22.5)	99,902 (56.7)
-	Male	77,692 (43.7)	1344 (77.5)	76,348 (43.3)
-	Unknown	3 (0.0)		3 (0.0)
Race/ethnicity, n (%)	Asian	3653 (2.1)	10 (0.6)	3643 (2.1)
-	Black	33,445 (18.8)	681 (39.3)	32,764 (18.6)
-	Hispanic	37,162 (20.9)	397 (22.9)	36,765 (20.9)
-	Other	3909 (2.2)	35 (2.0)	3874 (2.2)
-	Unknown	2011 (1.1)	6 (0.3)	2005 (1.1)
-	White	97,807 (55.0)	605 (34.9)	97,202 (55.1)
Language, n (%)	English	160,269 (90.0)	1650 (95.2)	158,619 (90.0)
-	Other/unknown	3947 (2.2)	9 (0.5)	3938 (2.2)
-	Spanish	13,771 (7.7)	75 (4.3)	13,696 (7.8)
Insurance, n (%)	Other/unknown	10,970 (6.2)	85 (4.9)	10,885 (6.2)
-	Private	89,015 (50.0)	209 (12.1)	88,806 (50.4)
-	Public	78,002 (43.8)	1440 (83.0)	76,562 (43.4)
Employment status, n (%)	Disabled	10,771 (6.1)	278 (16.0)	10,493 (6.0)
-	Employed	77,149 (43.3)	391 (22.5)	767,58 (43.5)
-	Not employed	35,004 (19.7)	991 (57.2)	34,013 (19.3)
-	Retired	43,803 (24.6)	49 (2.8)	43,754 (24.8)
-	Student	8861 (5.0)	11 (0.6)	8850 (5.0)
-	Unknown	2399 (1.3)	14 (0.8)	2385 (1.4)
Housing insecurity, n (%)	No	177,638 (99.8)	1696 (97.8)	175,942 (99.8)
-	Yes	349 (0.2)	38 (2.2)	311 (0.2)

Hx, history.

In the cohort with a history of incarceration, 52.4% were current smokers and 82.3% had ever smoked, compared with 16.49% and 43.8%, respectively, in the nonincarceration group. Furthermore, 65.8% of the incarcerated cohort reported current or former substance use, compared with 16.4% in the nonincarcerated group. Patients with a history of incarceration had a greater frequency of experiencing the use of restraint (6.5% vs 1.8%) and sedation (6.1% vs 1.7%). Regarding disposition from the EDs, patients with a history of incarceration had comparable rates of discharge (71.1% vs 72.7%) and admission (15.8% vs 17.5%) to those without such a history. However, those with a history of incarceration were more likely to elope (1.7% vs 0.5%) and leave against medical advice (1.1% vs 0.5%) (Table 2).

**Table 2:** Encounter specific information and care processes of patients with and without a history of incarceration in the ED.

Category		Overall	Any Hx of incarceration	No. Hx of incarceration
n		177,987	1734	176,253
ESI level, n (%)	1	1029 (0.6)	19 (1.1)	1010 (0.6)
-	2	31,760 (17.8)	529 (30.5)	31,231 (17.7)
-	3	86,686 (48.7)	698 (40.3)	85,988 (48.8)
-	4	45,638 (25.6)	436 (25.1)	45,202 (25.6)
-	5	10,907 (6.1)	46 (2.7)	10,861 (6.2)
-	Unknown	1967 (1.1)	6 (0.3)	1961 (1.1)
Restraint use, n (%)	No	174,772 (98.2)	1621 (93.5)	173,151 (98.2)
-	Yes	3215 (1.8)	113 (6.5)	3102 (1.8)
Sedation use, n (%)	No	174,913 (98.3)	1629 (93.9)	173,284 (98.3)
-	Yes	3074 (1.7)	105 (6.1)	2969 (1.7)
Disposition from ED, n (%)	AMA	852 (0.5)	19 (1.1)	833 (0.5)
-	Admit	31,162 (17.5)	274 (15.8)	30,888 (17.5)
-	Discharge	129,347 (72.7)	1233 (71.1)	128,114 (72.7)
-	Eloped	876 (0.5)	29 (1.7)	847 (0.5)
-	Expired	440 (0.2)	8 (0.5)	432 (0.2)
-	LWBS	1226 (0.7)	23 (1.3)	1203 (0.7)
-	Observation	10,708 (6.0)	130 (7.5)	10,578 (6.0)
-	Other/unknown	1969 (1.1)	7 (0.4)	1962 (1.1)
-	Transfer to another facility	1407 (0.8)	11 (0.6)	1396 (0.8)
Smoking history, n (%)	Never	90,489 (50.8)	209 (12.1)	90,280 (51.2)
-	Unknown	8805 (4.9)	60 (3.5)	8745 (5.0)
-	Yes	78,693 (44.2)	1465 (84.5)	77,228 (43.8)
Substance use history, n (%)	No	145,574 (81.8)	582 (33.6)	144,992 (82.3)
-	Unknown	2451 (1.4)	11 (0.6)	2440 (1.4)
-	Yes	29,962 (16.8)	1141 (65.8)	28,821 (16.4)
Presence of PCP, n (%)	No	4211 (2.4)	51 (2.9)	4160 (2.4)
	Yes	173,776 (97.6)	1683 (97.1)	172,093 (97.6)

AMA, against medical advice; ED, emergency department; ESI, emergency severity index; Hx, history; LWBS, left without being seen; PCP, primary care provider.

Using a multivariable logistic regression model for history of incarceration, we found significant associations with smoking and substance use history (2.45 [2.12-2.83], 2.31 [2.06-2.59]). Patients with known PCP had significantly lower aOR (0.66 [0.49-0.90]). Specific medical histories emphasized in prior literature were analyzed.^24^, ^25^, ^26^ Patients with a history of incarceration had higher aOR for depressive disorders, anxiety and fear-related disorders, suicidal ideation attempt/intention/harm, and schizophrenia spectrum and other psychotic disorders (1.82 [1.59-2.08], 1.25 [1.10-1.42], 3.03 [2.64-3.48], 1.81 [1.57-2.09]). Patients with hypertension and diabetes had significantly lower aOR (0.86 [0.76-0.98], 0.86 [0.76-0.98]). Patients with asthma, chronic kidney disease, acute myocardial infarction, and chronic obstructive pulmonary disease, and bronchiectasis did not have significantly different odds [0.86 (0.76-0.98), 0.91 (0.64-1.25), 0.80 (0.56-1.12), 1.12 (0.89-1.39)].

Regarding presentation, medical, trauma, and agitation chief concerns (1.00 [0.82-1.20], 1.12 [0.91-1.37], 1.49 [0.85-2.48]) were not significantly different, but patients with cognitive/neurologic, alcohol/drug, or psychiatric chief concerns (1.44 [1.12-1.82], 1.43 [1.15-1.76], 1.30 [1.04-1.61]) had significantly higher aORs. Although there were significantly different ORs between patients within and without a history of incarceration for both restraint and sedation usage (3.89 [3.19-4.70], 3.76 [3.06-4.57]), when adjusting for other features, included in Table 3 and Figure, there was no significant difference in aOR (1.26 [0.52-2.68], 0.85 [0.39-2.08]).

**Table 3:** Demographic covariates and unadjusted and adjusted odds ratios (ORs) for predicting history of incarceration.

Category		OR (univariable)	aOR (multivariable)
Age group (y)	18-24	-	-
-	25-34	3.99 (3.11-5.19)	2.14 (1.64-2.84)
-	35-44	4.84 (3.79-6.29)	2.45 (1.87-3.25)
-	45-54	4.61 (3.59-6.00)	2.68 (2.03-3.58)
-	55-64	3.38 (2.63-4.42)	1.96 (1.47-2.65)
-	65+	0.42 (0.30-0.58)	0.89 (0.59-1.34)
Sex	Female	-	-
	Male	4.51 (4.03-5.06)	4.04 (3.58-4.57)
Race ethnicity	White	-	-
-	Hispanic	1.75 (1.54-1.99)	1.50 (1.29-1.73)
-	Black	3.37 (3.02-3.77)	2.37 (2.09-2.69)
-	Asian	0.45 (0.22-0.79)	0.89 (0.43-1.61)
-	Other	1.47 (1.02-2.03)	1.61 (1.10-2.28)
Language	English	-	-
-	Spanish	0.53 (0.41-0.66)	0.62 (0.47-0.81)
-	Other	0.22 (0.11-0.40)	0.63 (0.30-1.16)
Employment	Employed	-	-
-	Not employed	5.69 (5.07-6.40)	2.80 (2.47-3.18)
-	Student	0.24 (0.13-0.42)	0.52 (0.26-0.92)
-	Retired	0.22 (0.16-0.29)	0.71 (0.48-1.03)
-	Disabled	5.18 (4.43-6.04)	2.08 (1.73-2.49)
-	Housing insecurity	12.68 (8.88-17.57)	1.41 (0.94-2.05)
Management	Restraint use	3.89 (3.19-4.70)	1.28 (0.53-2.72)
-	Sedation use	3.76 (3.06-4.57)	0.83 (0.38-2.02)
Health behaviors	Smoking history	6.98 (6.14-7.97)	2.67 (2.32-3.09)
-	Substance use history	9.84 (8.91-10.88)	2.45 (2.19-2.75)
-	Presence of PCP	0.80 (0.61-1.07)	0.70 (0.52-0.95)
Disposition from ED	Discharge	-	-
-	Admit	0.93 (0.81-1.06)	0.87 (0.75-1.01)
-	Observation	1.29 (1.07-1.54)	0.97 (0.79-1.19)
-	Expired	1.94 (0.88-3.65)	1.29 (0.55-2.62)
-	Transfer to another facility	0.83 (0.43-1.42)	0.69 (0.34-1.25)
-	AMA	2.39 (1.46-3.67)	1.24 (0.74-1.98)
-	LWBS	2.00 (1.28-2.97)	1.32 (0.82-2.01)
-	Eloped	3.59 (2.41-5.12)	1.65 (1.08-2.43)
-	Other	0.00 (0.00-0.01)	0.00 (0.00-0.02)
Chief concern	Medical CC	0.43 (0.39-0.47)	1.00 (0.83-1.21)
-	Trauma CC	0.89 (0.77-1.02)	1.16 (0.94-1.42)
-	Cognitive/neuro CC	1.09 (0.89-1.32)	1.46 (1.15-1.85)
-	Alcohol/drug CC	5.85 (5.03-6.78)	1.48 (1.20-1.83)
-	Psychiatric CC	5.15 (4.50-5.87)	1.30 (1.04-1.61)
-	Agitation CC	4.92 (3.03-7.51)	1.50 (0.86-2.49)
Past medical Hx	Depressive disorders	4.59 (4.17-5.04)	1.87 (1.63-2.13)
-	Anxiety and fear-related disorders	3.01 (2.73-3.32)	1.26 (1.11-1.44)
-	Suicidal ideation attempts intentional self-harm	14.81 (13.35-16.41)	3.14 (2.73-3.60)
-	Schizophrenia∖_spectrum∖_and∖_other∖_psychotic∖_disorders	12.71 (11.35-14.22)	1.82 (1.57-2.10)
-	Hypertension	0.69 (0.62-0.77)	0.87 (0.76-0.98)
-	Asthma	1.30 (1.16-1.45)	0.93 (0.82-1.06)
	Diabetes mellitus	0.76 (0.66-0.88)	0.81 (0.69-0.95)
-	Chronic kidney disease	0.59 (0.42-0.79)	0.92 (0.65-1.26)
-	Acute myocardial infarction	0.68 (0.48-0.93)	0.79 (0.55-1.11)
-	Chronic obstructive pulmonary disease and bronchiectasis	1.05 (0.86-1.28)	1.13 (0.90-1.40)

AMA, against medical advice; ED, emergency department; Hx, history; LWBS, left without being seen; PCP, primary care provider.

**Figure:**
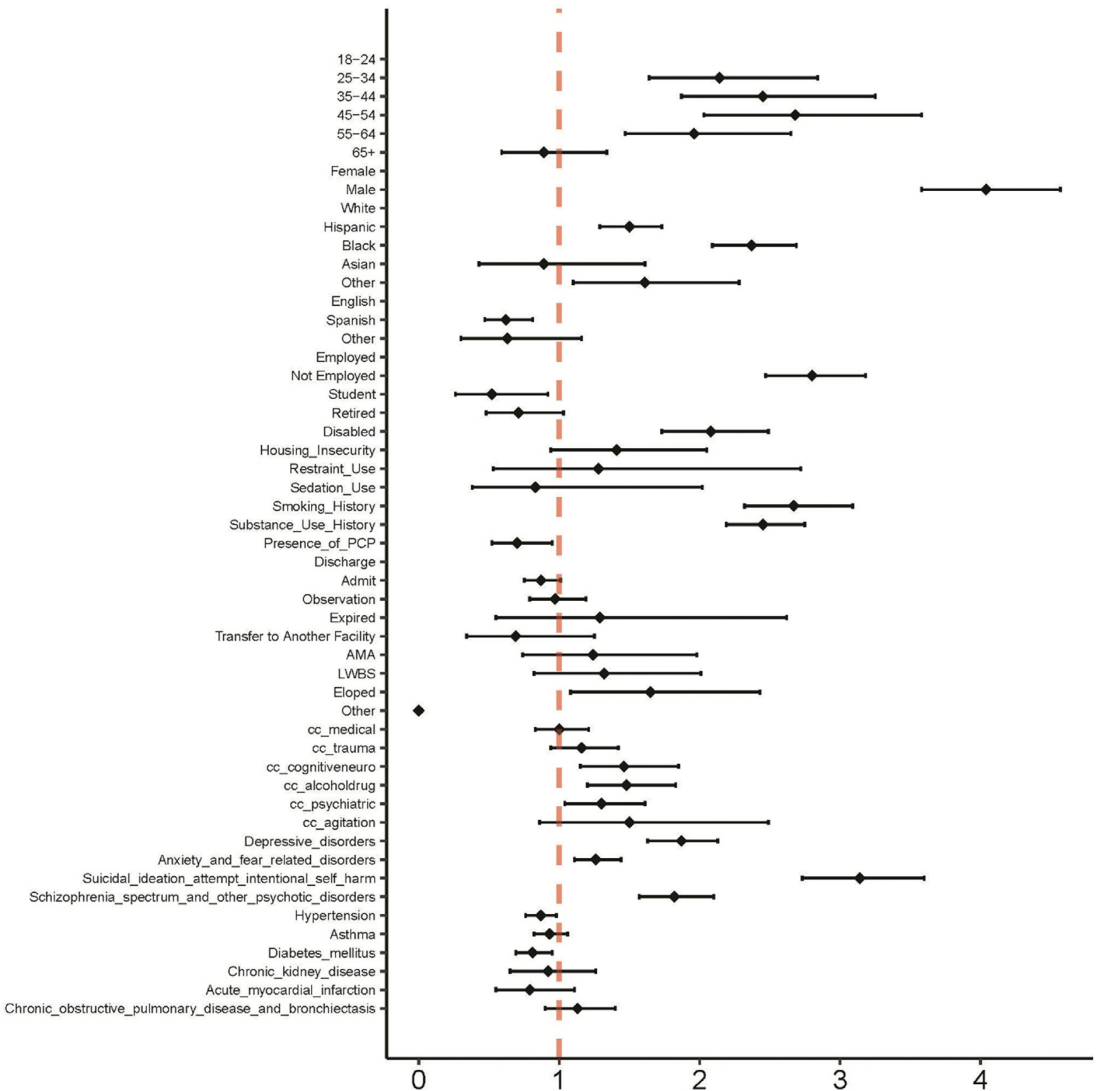
Forest plot of adjusted odds ratios for history of incarceration. AMA, against medical advice; LWBS, left without being seen; PCP, primary care provider.

## 4 Limitations

Although our model, with a positive predictive value of 95%, offers improved performance over previous methods for identification, the identification is limited by the accuracy of documentation in the ED due to the nature of a natural language processing-based method. In addition, our methodology identifies both prior history and current incarceration. Both patient and provider hesitancy to document incarceration status given the pervasive stigma of incarceration history can limit the accuracy of documentation and thus our method of identification. Greater nuance necessitates distinguishing characteristics of specific subcohorts within this group. Other data, including demographic characteristics, historical health information, and management metrics within the EDs, are also limited by the quality collected within the EHRs and available for extraction.

The analysis, confined to a 1-year period, provides a snapshot that may not fully capture longitudinal health care trends or the evolving landscape of social determinants of health. Despite these limitations, the study represents a significant step forward in the sensitive identification of incarceration history and in highlighting disparities in ED presentations and outcomes.

## 5 Discussion

Our analysis demonstrates the feasibility and utility of using LLMs to study the health system experience of people with histories of incarceration. We identified 1734 (0.97%) patients with a history of incarceration from a total of 177,987 unique ED patients, whose socio-demographic and clinical characteristics mirror national and regional estimates, demonstrating the external validity of our data. People with histories of incarceration were more likely to be of a racially minoritized population and report unemployment and disability, aligning with prior literature.^4^^,^^27^ This cohort demonstrated significant differences in smoking and substance use histories, with a majority being current smokers, suggesting the necessity for robust, integrated health and substance use treatment programs to be connected to the ED setting.^28^

Notably, a history of incarceration is negatively correlated with PCP utilization, which may explain the lower aOR or lack of significant differences in hypertension, asthma, diabetes, chronic kidney disease, myocardial infarction, chronic obstructive pulmonary disease, and bronchiectasis histories. This finding is not expected in comparison to prior literature and may be a reflection of poor documentation as a result of limited access to community health care in this population of patients with a history of incarceration.^7^^,^^29^^,^^30^ Psychiatric-related comorbidities had significantly greater aOR, as well as significantly higher aOR in presentations of cognitive/neurologic, alcohol/drug, and psychiatric chief concerns. These findings align with prior literature on currently incarcerated patients, but our study elucidates not only patients currently incarcerated but also patients with a prior history of incarceration specifically presenting to the ED.^31^, ^32^, ^33^

Patients with a history of incarceration had greater odds of elopement from the ED. In a patient population with historically poor access to health care, it is important to consider the long-term consequences of elopement on these patients, such as increased risk of suicidal behavior.^34^^,^^35^ In addition, although judicious use of restraints or sedation may be unavoidable, there are behavioral techniques or preemptive attention that may help circumvent these situations. Despite finding a higher proportional usage of restraints and sedation with a significantly greater unadjusted OR, our analysis did not demonstrate a significantly different aOR when controlling for other covariates such as chief concern. Additional analysis showed agitation, alcohol/substance-related, and psychiatric chief concerns had the greatest correlation with the usage of sedation and restraints which are notably the same chief concerns significantly correlated with a history of incarceration. This degree of collinearity—as well as the possibility that providers who determine the chief concern during triage may be aware of and influenced by patients’ histories of incarceration—may foreshadow the effect of incarceration on restraint and sedation usage in the ED. It is important to continue carefully considering the usage of restraints and sedation, especially with significant associations of restraints with harm (apnea and physical injuries) and sedation (arrhythmia, sedation, and hypotension).^22^^,^^36^

Future research should build on this work by expanding the data set across multiple years and employing methodologies that control for and investigate other metrics in the EHR to provide a more nuanced understanding of the health care needs and outcomes of formerly incarcerated patients. However, it is important to discuss the ethical considerations of utilizing such methods to study vulnerable subpopulations, specifically individuals who are currently or previously incarcerated. Hesitancy by both patients to disclose incarceration history as well as providers to include this information in their notes should be heavily considered when utilizing such models and drawing conclusions from such analysis. It is important to take special consideration and care when accessing such information. This study utilizes our developed LLM as a potential cohort identifier to conduct large, deidentified, analysis at a population level rather than a tool for specific identification at the individual level.

In this analysis, we established the potential and utility of leveraging for identifying patients with a history of incarceration as a feasible method of studying the care processes of a marginalized population without having to reveal their status to health system providers. Our exploration of care processes in the ED has identified potential points for intervention, including the provision of nicotine and substance use disorder treatment as well as greater awareness for patient-centered practices that may help address disparate rates of elopement. These disparities characterized across multiple axes within the hospital underscore how imperative it is for ED health care providers to develop tailored approaches based on the unique needs of patients with a history of incarceration.

## Author Contributions

RAT and KHW conceived the study and designed the analyses. VS and TH provided data engineering. TH and PO analyzed the data. TH, PO, VS, EW, and RAT drafted the manuscript, and all authors contributed substantially to its revision. RAT and KHW take responsibility for the paper as a whole.

## Funding and Support

This publication was made possible by the Yale School of Medicine Fellowship for Medical Student Research.

## Conflict of Interest

The authors declare that they have no known competing financial interests or personal relationships that could have appeared to influence the work reported in this paper.

## Supplementary Materials

Supplementary Appendix A1-A2
