# Supplementary material for: Characterizing Emergency Department Care for Patients With Histories of Incarceration
Source: J Am Coll Emerg Physicians Open. 2025 Jan 10;6(1):100022. doi: 10.1016/j.acepjo.2024.100022 (PMC11852703; doi:10.1016/j.acepjo.2024.100022)
Supplement: Supplementary Appendix A1-A2 [file mmc1.docx]

Appendix

| **Note Type** | **Frequency** |
| --- | --- |
| ED Notes | 158636 |
| ED Provider Notes | 156387 |
| Discharge Summary | 68909 |
| Case Management | 62677 |
| Psychiatry | 22504 |
| ED Observation Note | 9253 |
| ED Psychiatric Eval Note | 2008 |

A1. Frequency Counts of Note Types Extracted for Clinical-Longformer Identification of Incarceration Status

A2. Inclusion Criteria List for Substance Use History:

"Crack" cocaine; Alprazolam; Amphetamines; Amyl nitrate; Barbiturates; Benzodiazepines; BUP; Cannabis; Clonazepam; Cocaine; Codeine; Crystal Meth; Diazepam; DMT; Fentanyl; Hashish; Heroin; Hydrocodone; Hydromorphone; Ketamine; Kratom; lorazepam; LSD; Marijuana; MDMA (Ecstasy); Mescaline; Methamphetamines; Methylphenidate; Morphine; Nitrous oxide; Opium; Oxycodone; PCP; Percocet; Psilocybin; Salvia; Solvent inhalants; Speedball
